# Cannabinoid Hyperemesis Syndrome: A Case Study in a Tunisian Young Man

**DOI:** 10.1155/2021/6617148

**Published:** 2021-02-06

**Authors:** Haythem Yacoub, Hajer Hassine, Héla Kchir, Nadia Maamouri

**Affiliations:** ^1^Gastro-enterology B Department La Rabta Hospital, Tunis, Tunisia; ^2^Faculty of Medicine of Tunis, El Manar University, Tunis, Tunisia

## Abstract

The increasing prevalence of cannabis use in the world requires awareness of cannabis-related disorders such as cannabinoid hyperemesis syndrome. This syndrome includes cyclic episodes of nausea, vomiting, and the learned behavior of hot bathing in individuals with chronic cannabis use. We present the case of a suspected cannabinoid hyperemesis syndrome that required a review of the literature to retain the diagnosis. The following case illustrates how cannabinoid hyperemesis syndrome awareness may lead to the diagnosis.

## 1. Introduction

The cannabinoid hyperemesis syndrome (CHS) was first described in 2004. It occurs in individuals with chronic cannabis abuse and includes cyclic episodes of nausea, vomiting, and abdominal pain, symptoms' relief with hot showers or baths, and resolution with cannabis discontinuation [1]. The lack of the screening is the main reason that CHS is underdiagnosed [2]. The following case illustrates how the lack of CHS awareness may mask diagnosis [3]. It is important to have in mind this infrequently reported syndrome.

## 2. Case Report

**Figure 1:**
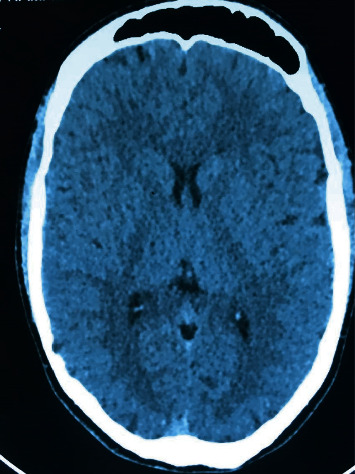
Normal cerebral CT scan.

**Figure 2:**
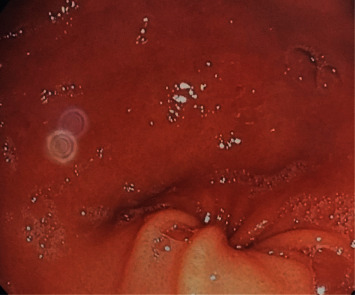
Congestive gastropathy.

We present the case of a 23-year-old male, with no significant medical history, who was admitted in our unit because of upper quadrant abdominal discomfort, recurrent nausea and vomiting, and weight loss of 4 kg in the last three months. During medical history, he revealed multiple emergency department visits (4 visits over a 12-week period) and two 24-hour hospitalizations for recurrent vomiting within this period. He also reported episodes lasting for several days characterized by severe nausea with vomiting between 8 and 10 times per day and abdominal pain. Episodes lasted approximately 3 days with full recovery between attacks. Attacks' frequency in our patient was 2 per month. In the emergency department, the patient was managed with treatment for dehydration including intravenous fluids. Antiemetics, including metoclopramide, were ineffective. His social history revealed daily cannabis use and occasional alcohol intake. The patient's mother stated that he began smoking cannabis at the age of 18 years and that he was smoking 6 cigarettes daily (3 g per day). On examination, the temperature was 37.2°C, the abdomen was tender on palpation of upper quadrants, blood pressure was 120/80 mm Hg, and pulse was 75 bpm. Neurological examination was negative for focal deficit. Liver function tests showed normal levels of alkaline phosphatase (ALP), gamma-glutamyl transferase (GGT), aspartate aminotransferase (AST), alanine aminotransferase (ALT), and total bilirubin. Other parameters including leukocytes, hemoglobin, platelet counts, renal function tests, basic metabolic panel, and glucose were normal. Abdominal ultrasound did not show gallbladder or pancreatic pathology. A cerebral CT scan was performed, and it was normal (Figure 1). Upper gastrointestinal endoscopy was performed and showed congestive gastropathy (Figure 2). *H. pylori* infection was detected on antrum and lesser curvature biopsies and was correctly treated with a bismuth-containing quadruple therapy. Cannabinoid hyperemesis syndrome was highly suspected: recurrent episodes of severe nausea and intractable vomiting, abdominal pain, and daily habitual use of cannabis.

Multiple sessions of supportive psychotherapy were provided to our patient by a psychiatrist, and he was encouraged to quit cannabis. Cognitive behavioral therapy helped our patient to identify contingencies of using behavior and developed relapse prevention and coping skills. A pharmacological treatment based on antidepressants and anxiolytics was proposed to our patient.

The patient reported less nausea, vomiting, and abdominal pain after two months of decreasing daily drug use and completely resolved after cessation of cannabis use (two months after starting the therapy). At a 7-month follow-up appointment, the patient was completely asymptomatic.

## 3. Discussion

Cannabinoid hyperemesis syndrome (CHS) was first described in the literature in 2004 and has increasingly appeared in multiple articles in the literature. However, it is presumed to be underdiagnosed because of its similarity to other diseases and limited research [1]. Patients presenting with CHS may not be forthcoming with their cannabis use history. The pathophysiology is not fully understood. Tetrahydrocannabinol is the most known active compound in cannabis; it is distributed throughout the central nervous system, the liver, the pancreas, and small intestine cannabinoid receptors, CB1 and CB2. CB1 is believed to be responsible for most of the clinical effects of cannabis use and CHS [4].

The increasing prevalence of cannabis use all over the world requires awareness of cannabis-related disorders including CHS. Nearly in all cases, several years of cannabis use were noticed before the onset of symptoms [5].

Cannabinoid hyperemesis syndrome is associated predominately with a male population who use cannabis daily or at least weekly and have used cannabis for at least one year [5]. To our knowledge, this is the first reported case in Tunisian literature. Four different phases have been identified in the literature: the prodromal, emetic, recovery, and interepisodic phases [6].

The symptoms of the prodromal phase occur most commonly in the morning with anorexia, nausea, and abdominal discomfort commonly reported as epigastric pain. Vomiting is not the major symptom during this phase [7, 8]. In this phase, the affected individuals may increase their cannabis consumption believing it will decrease their symptoms.

The second phase (emetic phase) is characterized by nausea, vomiting, flushing, diaphoresis, and diffuse abdominal pain lasting at least 24–48 hours [9]. Weight loss is common during this phase. Hot water showers have been reported to relieve symptoms. It is during this phase that most people present multiple times to the emergency department.

During the recovery phase, resolution of all symptoms occurs with return to the normal state and weight gain back to baseline [8]. Complete cessation of cannabis use is the only successful measure in ceasing symptoms. During the interepisodic phase, patients are comparatively free of symptoms.

The proposed CHS diagnostic criteria include stereotypical episodic vomiting resembling cyclic vomiting syndrome in terms of onset, frequency ≥3 episodes a year, cannabis use duration of more than one year preceding the onset of symptoms, frequency of use >4 times a week, on average, and finally, resolution of symptoms should follow a period of cessation from cannabis for a minimum of 6 months or at least equal to a duration that spans three typical cycles in an individual patient [10].

Sorensen et al. conducted a systematic review of the literature and calculated the frequency of these signs in 211 patients diagnosed with this syndrome and concluded that severe nausea or vomiting occurred in all patients. Weekly cannabis use was found in 97% of patients, and frequent hot water showers were found in 92% of patients [11]. In our patient, hot showers did not completely relieve symptoms.

Laboratory evaluation may demonstrate few abnormalities. Electrolyte irregularities and dehydration and renal failure may be significant during the hyperemetic stage because of frequent vomiting. However, it is not uncommon to have normal laboratory parameters because of the cyclic nature of CHS [6].

Multiple tests such as abdominal computed tomography (CT), cerebral CT scan, and upper gastroscopy are frequently performed to ensure that the pathological process does not exist. CHS awareness may reduce the number of unnecessary additional exams.

The patient is managed with treatment for dehydration, nausea, anxiety, and other medical issues which may include intravenous (IV) fluids, anxiolytics, and antipsychotics.

Treatment should also focus on cessation of cannabis use because currently, it is the only effective treatment. However, haloperidol intravenously or intramuscularly was found to have a good therapeutic effect for relief of nausea and vomiting. Capsaicin cream applied to the abdomen has a good effect on the same symptoms [5, 12]. Counseling for addiction should be included in the therapy plan.

The lack of screening is the main reason that CHS is underdiagnosed, and it is the most important tool in diagnosis [2].

## 4. Conclusion

Cannabinoid hyperemesis syndrome is a challenging diagnosis in patients who frequently use cannabis. It is important to educate cannabis users on all the potential consequences of its use. This public health message needs to be spread by all participants of the healthcare community. Adequate and thorough knowledge of the understanding of CHS is imperative for leading to the diagnosis and to provide adequate management.

## Data Availability

The data used to support the findings of this study are available from the corresponding author upon request.

## References

[1] Allen J. H., de Moore G. M., Heddle R., Twartz J. C. (2004). Cannabinoid hyperemesis: cyclical hyperemesis in association with chronic cannabis abuse. *Gut*.

[2] Hernandez J. M., Paty J., Price I. M. (2018). Cannabinoid hyperemesis syndrome presentation to the emergency department: a two-year multicentre retrospective chart review in a major urban area. *CJEM*.

[3] Hinton K. L., Chui J. S., McWhorter K. A., Jallad R. H., Siple J. F. (2016). Cannabinoid hyperemesis syndrome. *Annals of Pharmacotherapy*.

[4] Chanda D., Neumann D., Glatz J. F. C. (2019). The endocannabinoid system: overview of an emerging multi-faceted therapeutic target. *Prostaglandins, Leukotrienes and Essential Fatty Acids*.

[5] Lapoint J., Meyer S., Yu C. (2018). Cannabinoid hyperemesis syndrome: public health implications and a novel model treatment guideline. *Western Journal of Emergency Medicine*.

[6] Rehman A.-U., Pervaiz A., Narayan M., Saqib S. (2019). Cannabinoid hyperemesis syndrome - recognition, diagnosis and treatment. *Progress in Neurology and Psychiatry*.

[7] Simonetto D. A., Oxentenko A. S., Herman M. L., Szostek J. H. (2012). Cannabinoid hyperemesis: a case series of 98 patients. *Mayo Clinic Proceedings*.

[8] Richards J. R. (2018). Cannabinoid hyperemesis syndrome: pathophysiology and treatment in the emergency department. *The Journal of Emergency Medicine*.

[9] Hermes-Laufer J., Del Puppo L., Inan I., Troillet F. X., Kherad O. (2016). Cannabinoid Hyperemesis syndrome: a case report of cyclic severe hyperemesis and abdominal pain with long-term cannabis use. *Case Reports in Gastrointestinal Medicine*.

[10] Venkatesan T., Levinthal D. J., Li B. (2019). Role of chronic cannabis use: cyclic vomiting syndrome vs cannabinoid hyperemesis syndrome. *Neurogastroenterology & Motility*.

[11] Sorensen C. J., DeSanto K., Borgelt L., Phillips K. T., Monte A. A. (2017). Cannabinoid hyperemesis syndrome: diagnosis, pathophysiology, and treatment-a systematic review. *Journal of Medical Toxicology*.

[12] Kast K. A., Gershengoren L. (2018). Cannabinoid hyperemesis syndrome and the consulting psychiatrist. *Journal of Psychiatric Practice*.

